# Does high and intensive care reduce coercion? Association of HIC model fidelity to seclusion use in the Netherlands

**DOI:** 10.1186/s12888-020-02855-y

**Published:** 2020-09-29

**Authors:** A. L. Van Melle, E. O. Noorthoorn, G. A. M. Widdershoven, C. L. Mulder, Y. Voskes

**Affiliations:** 1 Department of Medical Humanities, Medical Faculty, Amsterdam University Medical Centers, location VUmc, F-wing, De Boelelaan 1089a, 1081 HV Amsterdam, The Netherlands; 2 Institute for Medical Ethics and History of Medicine, Ruhr University Bochum, Bochum, Germany; 3 GGNet Mental Health Centre, Apeldoorn, The Netherlands; 4 Parnassia Bavo Group, Rotterdam, The Netherlands; 5 Erasmus Medical Center, Rotterdam, The Netherlands; 6 Mental Healthcare Centre GGZ Breburg, Tilburg, The Netherlands; 7 Tilburg School of Social and Behavioral Sciences, Tranzo Scientific Center for Care and Welfare, Tilburg University, Tilburg, The Netherlands

**Keywords:** Seclusion, Coercion, Model fidelity, Psychiatry, High and intensive care (HIC)

## Abstract

**Background:**

A new inpatient care model has been developed in the Netherlands: High and Intensive Care (HIC). The purpose of HIC is to improve quality of inpatient mental healthcare and to reduce coercion.

**Methods:**

In 2014, audits were held at 32 closed acute admission wards for adult patients throughout the Netherlands. The audits were done by trained auditors, who were professionals of the participating institutes, using the HIC monitor, a model fidelity scale to assess implementation of the HIC model. The HIC model fidelity scale (67 items) encompasses 11 domains including for example team structure, team processes, diagnostics and treatment, and building environment. Data on seclusion and forced medication was collected using the Argus rating scale. The association between HIC monitor scores and the use of seclusion and forced medication was analyzed, corrected for patient characteristics.

**Results:**

Results showed that wards having a relatively high HIC monitor total score, indicating a high level of implementation of the model as compared to wards scoring lower on the monitor, had lower seclusion hours per admission hours (2.58 versus 4.20) and less forced medication events per admission days (0.0162 versus 0.0207). The HIC model fidelity scores explained 27% of the variance in seclusion rates (*p* < 0.001). Adding patient characteristics to HIC items in the regression model showed an increase of the explained variance to 40%.

**Conclusions:**

This study showed that higher HIC model fidelity was associated with less seclusion and less forced medication at acute closed psychiatric wards in the Netherlands.

## Background

The use of seclusion in psychiatry is highly problematic. Effects on reducing stimuli and creating a context for calming the patient, which are often mentioned as a reason for secluding an agitated patient, have not been demonstrated [1–4]. On the other hand, negative experiences and traumatizing effects have been shown [5, 6]. In acute adult psychiatry in the Netherlands, seclusion use has been an issue of debate over the past twenty years. The Dutch Government invested heavily in seclusion reduction between 2006 and 2012 [7–9]. A national program was started, aiming at reduction of seclusion by 10% a year, without substitution by other coercive measures, including forced medication. This aim was underlined by the Dutch ministry of Health, Welfare and Sport in a letter to the House of Representatives in 2012 [10]. Hospitals were provided with funding to improve involuntary care and to reduce seclusion. As part of this program, several interventions have been developed and implemented [11, 12]. The effects of some of these initiatives have been studied [8, 9]. The overall result was a reduction of the number and duration of seclusion of 41 and 30% respectively between 2008 and 2013 [8, 13]. Yet, not all institutions were successful, and some even showed an increase of seclusion rates [8]. Moreover, results from the national seclusion reduction programs showed a relative increase of forced medication by 81% between 2011 and 2013 suggesting substitution of seclusion by forced medication [8]. Long term follow-up data confirmed this impression [14, 15].

From several studies over the last decade we know comprehensive approaches in the reduction of seclusion and restraint to be substantially more effective than less comprehensive approaches [14, 16–20]. In order to further reduce seclusion and improve quality of care, from 2012 onwards a new comprehensive care model was developed for acute inpatient mental healthcare: High and Intensive Care (HIC) [21]. The HIC model combines new organization of care with a new care approach. The HIC model integrates the medical model and the recovery model and focuses on contact and crisis prevention and continuity of care between outpatient treatment and acute admission wards. The model is widely adopted in Dutch mental healthcare; a large majority of healthcare institutions have reorganized acute care and built new HIC wards. On these wards, patients are admitted for a maximum of 3 weeks, when outpatient treatment is no longer sufficient and admission to a closed setting is necessary. The HIC model aims at a reduction of coercive measures by improving healthcare practice using evidence- and practice-based approaches [21].

The HIC model focuses on hospitality at admission, care planning and risk assessment. Within the HIC ward a distinction is made between a “high-care function” and an “intensive-care function”. Initially, patients are admitted to the High Care (HC) section, consisting of single patient rooms, living areas and a comfort room. One-to-one care is given either at the HC section, or, depending on the severity and nature of the crisis, at the Intensive Care (IC) section. The IC section consists of several Intensive Care-Units (ICUs) with an individual bedroom and living area and High Security Rooms (HSRs). The purpose of the ICU is to provide one-to-one care in a separate area, without contact with other patients on the HC, while avoiding seclusion in a HSR for as long as possible.

The HIC model implies a set of quality criteria, described in the HIC monitor [21]. The monitor contains various domains, including team structure, team processes, diagnostics and treatment, and building environment. We hypothesized that a higher fidelity to the HIC model, as expressed in higher total scores on the monitor, to be associated with less coercion use (seclusion and forced medication).

This article presents the associations between HIC model fidelity and seclusion rates in acute psychiatric wards in the Netherlands. We aim to answer three research questions.

1. Is HIC model fidelity associated with seclusion rates?
2. Is HIC model fidelity associated to substitution of seclusion by forced medication?
3. How much variance of seclusion rates is explained by the HIC monitor scores taking patient characteristics into account?

## Methods

### Setting

By 2014, 84% of Dutch mental healthcare institutes with closed acute admission wards had adopted the HIC approach and had started to implement the HIC model. This study was carried out in 2014 in 32 closed acute admission wards for adult patients of 18 mental healthcare institutions throughout the Netherlands. These institutions all provided inpatient and outpatient services and differed in size of catchment area.

### Instruments

#### HIC monitor

The HIC monitor [21] is a model fidelity scale to measure the implementation level of the HIC model. It consists of 67 items, divided into 11 domains: (I) team structure, (II) team processes, (III) diagnostics, treatment, and treatment interventions, (IV) organization of care, (V) monitoring, (VI) professionalization, (VII) the Psychiatric Hospitals Compulsory Admissions Act (BOPZ), (VIII) the electronic health record, (IX) healing environment, (X) safety; and (XI) evaluation of and feedback on coercion. Wards were audited, using the HIC monitor. The items are scored on a 5-points scale ranging from 1 (not implemented) to 5 (fully implemented) by trained auditors, who were professionals of the participating institutes. Audits consisted of a full day of interviews with staff and patients, examination of health records and observation of the ward and multidisciplinary meetings in which staff discussed care for individual patients. The HIC monitor has been validated, resulting in minor changes [21] showing reasonably good interrater reliability and satisfactory content and construct validity. In this study, the old version of the monitor was used.

#### Assessing coercive measures

For a full year (2014) data on seclusion and forced medication were collected using the Argus rating scale [22]. Four types of coercive measures are included in the scale; seclusion, physical restraint, mechanical restraint and forced medication [23].

In the current study we used seclusion as main outcome. Seclusion was defined as the seclusion of a patient in a specifically designated room that has been approved by the health authority. The use of forced medication was used as a secondary outcome variable. For manual restraint by means of ‘holding’ is sporadically applied at some wards, and mechanical restraint is hardly used in acute adult psychiatric wards, restraint was not included in this study. In the Argus analysis model, coercive measures are identified as counters, and patient and ward characteristics as well as admission time as denominators [24].

#### Coercive measures (counters)

At a day-to-day level exact data were collected concerning the frequency and time spent in seclusion and the number of times forced medication was used. Hours spent in seclusion as well as number of events of forced medication were the counters [13].

#### Patient characteristics and admission time (denominators)

To understand the association between the HIC monitor scores and seclusion use we corrected for patient characteristics including age, gender, marital status, and diagnosis. Diagnoses were categorized in the main groups of the DSM-IV-TR [8]. These were included in the database next to admission time. The database was organized at the level of a single admission per record. The HIC monitor scores were given at ward level. This information was repeated in all patient admission records of a single ward. A readmitted patient could occur more than once in the database. Time spent in seclusion as compared to admission time was identified as outcome measure.

### Analysis

#### Association of HIC monitor scores and seclusion

First, we divided the wards into two groups based on the median HIC monitor score at the audits: wards with relatively high and low scores on the HIC monitor. We tested if a high or a low median sore on the HIC monitor was associated with a high or low use of seclusion or forced medication. Also, this allowed gaining an impression of substitution, which is the case when low seclusion figures are associated with high forced medication figures.

#### Associations of HIC monitor scores and seclusion, controlling for patient characteristics

Second, a multilevel logistic regression analysis was performed to understand the association of patient characteristics and scores on the HIC monitor with the use of seclusion in the wards. The proportion of time in seclusion as compared to admission time was modeled by a multilevel Generalized Linear Latent and Mixed Model (GLLAMM) module. First, a binomial distribution with the logistic link function (in short: multilevel Logistic regression) was used to relate independent variables to outcome variables. This technique can be extended from modeling dichotomous outcome variables to modeling proportions as outcome variables and we used the latter option [25]. A patient’s admission was identified as level 1, the patient as level 2, the ward as level 3. Model fit was determined at each step of the analysis by means of increase in McFadden’s R square [26]. Patient characteristics and HIC monitor findings could be seen as predictors, modifiers or confounders of seclusion use as outcome.

This analysis was performed in three steps. First, we investigated the direct association of the HIC monitor total scores with seclusion. Second, we investigated the association of patient characteristics and diagnosis with seclusion. Third, the HIC monitor total and item scores, patient characteristics and diagnosis were combined in a full model. For this last step, relevance of variables was identified by means of the criterion of Hosmer and Lemeshow [27]. This criterion suggests including only variables with a p – value of less than 0.2 in a next step of the model. In understanding the findings, the variables with a significant association to outcome as well as to the explained variance as expressed by the McFadden’s r^2^ are emphasized. The McFadden’s r^2^ provides an impression of the explained variance at full scale level. The multilevel analysis was performed in SPSS version 25 and checked in STATA version 12.

## Results

Table 1 presents the association of the total score on the HIC monitor with seclusion hours, hours seclusion per admission hours and number of forced medication events per admission days. It shows that wards scoring high on the HIC monitor have lower seclusion hours and less forced medication events than wards scoring low on the HIC monitor (hours of seclusion per admission hours was 0.0258 for the high scoring wards opposed to 0.0420 for the low scoring wards; medication events per admission days were 0.0162 for the high scoring wards opposed to 0.207 for the low scoring wards). We also observed wards with high seclusion exposure, also had higher forced medication exposure figures. We did not observe any evidence for substitution (more forced medication against less seclusion), even when observing the data at an institutional level.

**Table 1:** Differences between wards scoring high and low on the HIC monitor

HIC score	N wards	Seclusion hours	Number of Seclusion Incidents	Hours seclusion per admission hours**	Enforced Medication	Medication Events per admission days*
High > 184	17	40,476 h	690	2.58	538	0.0162
Low < 184	16	76,847 h	1404	4.20	1030	0.0207

*Significant differences student t test *p* < 0.05

**Significant differences student t test *p* < 0.001

Table 2 presents the findings of the multilevel regression analysis calculating the association between seclusion use as outcome and patient characteristics and the HIC monitor score as predictors of seclusion. The first two columns of Table 2 show HIC monitor total scores, as well as the percentages of patient characteristics in the total population and in the secluded patients. The next columns present the findings of the multilevel regression. The final columns present the findings of the multilevel analysis with the HIC monitor as well as the patient characteristics and diagnoses in the full model.

**Table 2:** Associations between the HIC monitor and patient characteristics with seclusion analyzed by means of multilevel analysis

*N=*			Basic frequencies		multivariable blockwise model					multivariable final model				R^2^	R^2^
			% or mean All patients	% or mean secluded											
					Ex (b)	95 CI ex (b)		p	R^2^	Ex (b)	95 CI ex (b)		p		
			6068	1058											
	HIC Total score				0.98	0.96	0.99	< 0.001	0.27					0.27	0.4
Patient characteristics	Demographics	Age**	41.1	37.1	0.97	0.96	0.98	0.000	0.17	0.97	0.96	0.98	0.000	0.27	
		Male**	56.1%	67.4%	1.78	1.42	2.26	0.000		1.75	1.38	2.21	0.000		
		Partner*	45.6%	41.9%	0.76	0.61	0.95	0.014		0.82	0.66	1.02	0.088		
	Diagnosis	No diagnosis**	18.4%	23.4%	1.97	1.41	2.77	0.000	0.27	1.59	1.18	2.15	0.002		
		Adjustment disorder	6.7%	5.5%											
		Anxiety disorder**	6.9%	3.8%											
		Depression**	10.6%	5.2%											
		Bipolar	8.9%	10.5%	1.75	1.15	2.64	0.008		1.81	1.22	2.68	0.003		
		Psychosis	15.2%	16.3%	1.62	1.12	2.34	0.009		1.39	1.01	1.92	0.043		
		Schizophrenia	10.5%	12.3%	1.82	1.24	2.68	0.002		1.50	1.06	2.12	0.020		
		Organic disorder	1.1%	0.9%%	2.23	0.96	5.17	0.060		3.08	1.72	7.47	0.013		
		Drug abuse	23.8%	23.9%	1.64	1.19	2.28	0.003							
		Developmental disorder & Autism	4.0%	3.9%											
		Intellectual Disability	2.0%	2.3%											
		Personality disorder**	26.5%	20.7%											
	HIC Items ^a^														

^a^ Presented in online table

**P < 0.001

* *P* < 0.05

The investigated institutions had a total of 7126 patients admitted at their HIC wards. Of these patients, 1058 (14.8%, range over wards 2.5–35.8%, 95% CI = 3.5–30.5%) were secluded. Higher scores on the HIC monitor were associated to less seclusion use (Ex(b) = 0.98, *P* < 0.001). Concerning patient characteristics young age, male gender, having no final diagnosis, bipolar disorder, psychosis, schizophrenia and organic disorder were associated with increased seclusion use. Having a partner was associated to less seclusion use. The explained variance of the HIC total score was 27%; the explained variance with respect to the patient characteristics was also 27%. Combining the full model of patient characteristics with HIC total score and items resulted in an explained variance of 40%. An additional file shows these results in more detail [see Additional file 1].

## Discussion

Wards that scored high on the HIC monitor, displaying higher HIC model fidelity and implementation of the HIC model, showed lower seclusion rates than wards that scored low on the HIC monitor. This shows that implementation of the HIC model contributes to the reduction of seclusion at acute closed psychiatric wards. Moreover, wards that scored high on the HIC monitor also showed low rates of forced medication, indicating that substitution of seclusion by forced medication did not occur. Confounding by patient compilation was ruled out using the multilevel analyses including patient characteristics.

The HIC monitor total score was associated to less seclusion use showing an explained variance of around 27%. The influence of patient factors on the use of seclusion is consistent with earlier studies, also showing an explained variance of around 27% [13]. When combined in a full model covering both HIC monitor total score and items and patient characteristics, a substantial increase in explained variance was visible. The explained variance by the full model increased to 40% which is a relatively high figure [26, 28]. McFadden’s R2 is a statistic indicating the approximate explained variation in logistic regression [26, 29]. A value under 20% indicates low explained variance, a value between 20 and 30% is a reasonable result, and a value above 40% designates a good level of explained variance [26, 28]. We may conclude that seclusion use is predicted by both the HIC model as well as patient characteristics.

This study investigated the association between the HIC monitor total score and items and the use of seclusion and forced medication. The association of seclusion and medication rates with domains and items within the HIC monitor is presented in the online appendix. These associations are less relevant than the overall score on the HIC monitor. The HIC model is a formative model in which the items and domains jointly represent the compliance with the HIC model [21, 30]. The structure of the HIC monitor has the form of a taxonomy that enables the user to measure compliance with the HIC model and thus provides a quality check on care being delivered. The domains within the model were not constructed by means of factor analysis but a priori based on the content of the items, as the strength of the HIC monitor lays in its function as a checklist to guide the process of implementation of the HIC model.

Our study included several mental healthcare institutions at one moment in time. This does not enable conclusions about the development within institutions. A recent study on reduction of seclusion in two institutions showed a substantial reduction of seclusion rates after implementation of the HIC methodology in one institution, while the other institution in which the HIC model was not actively implemented, showed less reduction of seclusion [14, 15]. For the time being, the longitudinal findings are anecdotal and originating from a small amount of institutions. It might be the case that these institutions support publication of their seclusion rates, which may imply a selection bias. In an international perspective, seclusion rates from institutions which allow for publication are lower than rates in otherwise similar institutions [31, 32]. Currently, the HIC model is only being implemented in the Netherlands. This precludes international comparison.

### Strengths and limitations

A strength of this multicenter study is the scope, as 21 mental healthcare institutions and 38 wards throughout the Netherlands were included. This allowed us to investigate the effects of the policy expressed in the HIC model on a large scale, as advised by Verlinde et al. (2016). Moreover, findings are based on 11,425 admissions of 7126 patients, making this study one of the largest studies looking into both patient and ward characteristics [33, 34]. Also, this study provides a confirmation of the construct validity of the HIC monitor as operationalization of the compliance to the HIC model and strengthens its psychometrical properties.

The study was not without limitations. First, the same data used to validate the HIC monitor were used in this study. However, since the instrument was already developed before this time and minor revisions were done to the HIC monitor after validation, the influence on the data is minimal [21]. Second, the analyses were done on cross-sectional data. In order to get insight into the causal effects of the HIC model on seclusion rates, experimental studies are needed.

## Conclusion

This study shows that the HIC model, combining interventions in a structured way, is associated with a reduction of seclusion at acute closed psychiatric wards in the Netherlands. Moreover, there is no indication of substitution of seclusion by forced medication when working according to HIC principles. The HIC model, combined with patient characteristics, has a high explained variance regarding seclusion use. As this study measured the association between HIC scores and seclusion rates in a cross-sectional way for 1 year, a follow up of developments over time is needed.

## Supplementary information

**Additional file 1.** This pfd-file includes the results of the multilevel analysis on item level.

## Data Availability

Supporting Information (S1 Data) accompanying this paper is available through OSF Repository (DOI 10.17605/OSF.IO/DK5JS). The file HICandcoercion2014.sav contains the underlying data for data sharing purposes. The first line of the database formatted in Excel contains the variable labels. Please contact the second author on e.noorthoorn@ggnet.nl when data are pooled.

## Abbreviations

DSM-IV-TR: Diagnostic and Statistical Manual of Mental Disorders - Fourth Edition - Text Revision
GLLAMM: Generalized Linear Latent and Mixed Model
HC: High Care
HIC: High and Intensive Care
HSR: High Security Room
IC: Intensive Care
ICU: Intensive Care Unit
SPSS: Statistical Package for the Social Sciences
STATA: Statistics And Data
Wet BOPZ: Wet Bijzondere Opname Psychiatrische Ziekenhuizen (Psychiatric Hospitals Compulsory Admissions Act)
