# Supplementary material for: Does high and intensive care reduce coercion? Association of HIC model fidelity to seclusion use in the Netherlands
Source: BMC Psychiatry. 2020 Sep 29;20:469. doi: 10.1186/s12888-020-02855-y (PMC7523051; doi:10.1186/s12888-020-02855-y)
Supplement: Supplementary file 1 — Additional file 1. This pfd-file includes the results of the multilevel analysis on item level. [file 12888_2020_2855_MOESM1_ESM.docx]

**Additional File 1. Table 3: Multi level analysis patient characteristics and HIC Monitor (detailed presentation for internet)**

|  | |  | % or mean | univariable associations | | | | multivariable blockwise model | | | | multivariable final model | | | | R^2^ | R^2^ | R^2^ |
| --- | --- | --- | --- | --- | --- | --- | --- | --- | --- | --- | --- | --- | --- | --- | --- | --- | --- | --- |
|  | | Item |  | Ex (b) | 95 CI ex (b) | | p | Ex (b) | 95 CI ex (b) | | p | Ex (b) | 95 CI ex (b) | | p |  |  |  |
| Patient characteristics | Demo-graphics | Age | 40,6 | 0.97 | 0.96 | 0.98 | 0.001 | 0.97 | 0.96 | 0.98 | 0.001 | 0.97 | 0.96 | 0.98 | 0.001 | 0.17 | 0.27 | 0.40 |
|  |  | Male | 57,3% | 1.86 | 1.48 | 2.45 | 0.001 | 1.79 | 1.42 | 2.26 | 0.001 | 1.79 | 1.40 | 2.29 | 0.001 |  |  |  |
|  |  | Partner | 45,1% | 0.79 | 0.64 | 0.97 | 0.023 | 0.76 | 0.61 | 0.94 | 0.014 |  |  |  |  |  |  |  |
|  | Diagnosis | No diagnosis | 18,9% | 1.30 | 1.02 | 1.65 | 0.032 | 1.97 | 1.41 | 2.77 | 0.001 | 1.84 | 1.25 | 2.68 | 0.002 | 0.27 |  |  |
|  |  | Adjustment disorder | 6,6% | 0.78 | 0.49 | 1.22 | 0.288 |  |  |  |  |  |  |  |  |  |  |  |
|  |  | Anxiety disorder | 6,6% | 0.64 | 0.39 | 1.05 | 0.079 |  |  |  |  |  |  |  |  |  |  |  |
|  |  | Depression | 10,0% | 0.51 | 0.32 | 0.79 | 0.003 |  |  |  |  |  |  |  |  |  |  |  |
|  |  | Bipolar | 9,1% | 1.11 | 0.79 | 1.55 | 0.543 | 1.75 | 1.16 | 2.64 | 0.008 | 1.69 | 1.07 | 2.85 | 0.023 |  |  |  |
|  |  | Psychosis | 15,3% | 1.02 | 0.77 | 1.34 | 0.886 | 1.62 | 1.12 | 2.34 | 0.009 |  |  |  |  |  |  |  |
|  |  | Schizophrenia | 10,7% | 1.17 | 0.86 | 1.59 | 0.315 | 1.82 | 1.24 | 2.68 | 0.002 |  |  |  |  |  |  |  |
|  |  | Organic disorder | 4,0% | 1.46 | 0.64 | 3.28 | 0.360 |  |  |  |  |  |  |  |  |  |  |  |
|  |  | Drug abuse | 23,9% | 1.05 | 0.83 | 1.33 | 0.626 | 1.64 | 1.19 | 2.28 | 0.003 |  |  |  |  |  |  |  |
|  |  | Developmental disorders & Autism | 4,0% | 1.08 | 0.66 | 1.79 | 0.740 |  |  |  |  |  |  |  |  |  |  |  |
|  |  | Intellectual Disability | 2,1% | 1.33 | 0.71 | 2.51 | 0.360 |  |  |  |  |  |  |  |  |  |  |  |
|  |  | personality disorder | 25,8% | 0.76 | 0.60 | 0.98 | 0.037 |  |  |  |  |  |  |  |  |  |  |  |
| HIC Monitor | Team structure | Small caseload: day shift | 3.16 (1.22) | 1.00 | 0.91 | 1.10 | 0.869 | 1.17 | 1.03 | 1.33 | 0.013 | 1.36 | 1.16 | 1.58 | 0.001 | 0.34 | 0.37 |  |
|  |  | Small caseload: evening shift | 2.92 (1.24) | 0.99 | 0.90 | 1.08 | 0.848 |  |  |  |  |  |  |  |  |  |  |  |
|  |  | Small caseload: night shift | 2.21 (0.84) | 0.96 | 0.85 | 1.08 | 0.579 |  |  |  |  |  |  |  |  |  |  |  |
|  |  | Stepped care | 3.68 (1.38) | 0.99 | 0.92 | 1.07 | 0.950 | 0.73 | 0.65 | 0.81 | 0.001 | 0.69 | 0.58 | 0.82 | 0.001 |  |  |  |
|  |  | Staff coverage | 4.55 (0.65) | 1.53 | 1,31 | 1,80 | 0.000 | 1.73 | 1.38 | 2.17 | 0.001 | 1.75 | 1.31 | 2.32 | 0.001 |  |  |  |
|  |  | Team | 2.45 (1.65) | 0.98 | 0,92 | 1.04 | 0,576 |  |  |  |  |  |  |  |  |  |  |  |
|  |  | Psychiatrists | 2.66 (1.48) | 0.98 | 0,92 | 1.04 | 0,560 |  |  |  |  |  |  |  |  |  |  |  |
|  |  | Psychologists | 1.21 (0.58) | 1.49 | 1.21 | 1.83 | 0.000 | 2.01 | 1.45 | 2.79 | 0.001 | 2.93 | 2.05 | 4.19 | 0.001 |  |  |  |
|  |  | Nursing specialists | 1.84 (1.35) | 0.87 | 0,81 | 0,94 | 0,000 | 0.74 | 0.67 | 0.82 | 0.001 | 0.64 | 0.54 | 0.75 | 0.001 |  |  |  |
|  |  | Addiction experts | 1.82 (1.61) | 0.96 | 0,90 | 1,02 | 0,233 | 0.82 | 0.74 | 0.91 | 0.001 | 0.84 | 0.75 | 0.96 | 0.007 |  |  |  |
|  |  | Peer providers | 1.42 (0.95) | 1.12 | 0,99 | 1.27 | 0.055 |  |  |  |  |  |  |  |  |  |  |  |
|  |  | Activity supervisors | 2.68 (1.48) | 1.24 | 1.15 | 1.33 | 0.000 | 1.13 | 1.03 | 1.24 | 0.006 |  |  |  |  |  |  |  |
|  |  | Activity program | 2.58 (1.48) | 1.06 | 0.96 | 1.17 | 0.195 |  |  |  |  |  |  |  |  |  |  |  |
|  |  | Supervisors/team leaders | 3.29 (1.14) | 0.91 | 0.83 | 1.00 | 0.063 | 1.13 | 1.01 | 1.27 | 0.030 |  |  |  |  |  |  |  |
|  |  | Extra disciplines | 3.61 (1.03) | 0,89 | 0.78 | 0.99 | 0.048 |  |  |  |  |  |  |  |  |  |  |  |
|  |  | Team structure |  |  |  |  |  | 1.00 | 0.97 | 1.03 | 0.709 |  |  |  |  |  |  |  |
|  | Team processes | Vision | 2.37 (1.40) | 1.02 | 0.94 | 1.10 | 0.515 |  |  |  |  |  |  |  |  | 0.36 |  |  |
|  |  | Hospitality and presence | 3.03 (1.08) | 1.02 | 0.93 | 1.12 | 0.553 |  |  |  |  |  |  |  |  |  |  |  |
|  |  | Attitude/treatment | 2.71 (1.21) | 0.99 | 0.90 | 1.08 | 0,845 |  |  |  |  |  |  |  |  |  |  |  |
|  |  | Coordination of care: at admission | 2.03 (1.28) | 0.93 | o,86 | 1.02 | 0.088 | 0.75 | 0.67 | 0.83 | 0.001 |  |  |  |  |  |  |  |
|  |  | Coordination of care: every 3 weeks | 2.92 (1.76) | 1.01 | 0.95 | 1.07 | 0.585 |  |  |  |  |  |  |  |  |  |  |  |
|  |  | Coordination of care: at discharge | 2.92 (1.63) | 1.03 | 0.97 | 1.10 | 0.280 |  |  |  |  |  |  |  |  |  |  |  |
|  |  | Digital whiteboard | 1.87 (1.31) | 1.24 | 1.12 | 1.38 | 0.000 | 1.44 | 1.27 | 1.63 | 0.001 |  |  |  |  |  |  |  |
|  |  | Consultation intensive care ward | 2.97 (1.87) | 1.08 | 1.02 | 1.14 | 0.003 |  |  |  |  |  |  |  |  |  |  |  |
|  |  | Consultation intensive care unit | 1.66 (1.40) | 1.11 | 1.03 | 1.20 | 0.006 | 1.16 | 1.07 | 1.26 | 0.001 |  |  |  |  |  |  |  |
|  |  | Consultation HSR | 1.55 (1.27) | 1.13 | 1.04 | 1.23 | 0.003 |  |  |  |  |  |  |  |  |  |  |  |
|  |  | Team processes |  |  |  |  |  |  |  |  |  |  |  |  |  |  |  |  |
|  | Diagnostics, treatment and treatment intervention | Guidelines | 3.53 (1.27) | 1.08 | 0.99 | 1.17 | 0.057 | 1.20 | 1.06 | 1.35 | 0.002 |  |  |  |  | 0.39 |  |  |
|  |  | Early diagnostics at admission | 4.47 (1.03) | 1.05 | 0.94 | 1.17 | 0.336 |  |  |  |  |  |  |  |  |  |  |  |
|  |  | Copy of treatment plan | 2.39 (1.62) | 1.14 | 1.07 | 1.22 | 0.000 | 1.14 | 1.06 | 1.23 | 0.001 | 1.29 | 1.17 | 1.43 | 0.001 |  |  |  |
|  |  | General examination: history | 2.92 (1.55) | 1.02 | 0.96 | 1.09 | 0.370 |  |  |  |  |  |  |  |  |  |  |  |
|  |  | General examination: medical | 4.03 (1.38) | 0.96 | 0.89 | 1.04 | 0.396 | 0.81 | 0.71 | 0.91 | 0.001 | 0.62 | 0.54 | 0.72 | 0.001 |  |  |  |
|  |  | Risk assessment | 2.58 (1.64) | 1.02 | 0.96 | 1.09 | 0.420 |  |  |  |  |  |  |  |  |  |  |  |
|  |  | Conflict control and personal safety | 4.37 (1.24) | 1.01 | 0.93 | 1.10 | 0.770 |  |  |  |  |  |  |  |  |  |  |  |
|  |  | Early and emergency medication | 3.47 (1.41) | 0.96 | 0.89 | 1.04 | 0.408 | 0.81 | 0.73 | 0.89 | 0.001 |  |  |  |  |  |  |  |
|  |  | Psycho-education | 2.47 (0.78) | 0.89 | 0,79 | 1.02 | 0.089 | 0.84 | 0.73 | 0.96 | 0.016 |  |  |  |  |  |  |  |
|  |  | Somatic screening | 3.42 (0.89) | 1.20 | 1.07 | 1.34 | 0.001 0.001 0.001 |  |  |  |  |  |  |  |  |  |  |  |
|  |  | Dual diagnosis | 1.74 (1.09) | 1.14 | 1.01 | 1.28 | 0.029 0.0010.029 | 1.17 | 1.02 | 1.36 | 0.024 |  |  |  |  |  |  |  |
|  |  | Family interventions | 3.18 (1.06) | 1.14 | 1.141 | 1.38 | 0.001 | 1.51 | 1.32 | 1.71 | 0.001 | 1.23 | 1.03 | 1.47 | 0.001 |  |  |  |
|  |  | Diagnostics, treatment and intervention | |  |  |  |  | 1.03 | 1.01 | 1.05 | 0.014 | 1.02 | 1.01 | 1.04 | 0.001 |  |  |  |
|  | admission | Admission and discharge criteria | 3.21 (1.66) | 1.07 | 1.00 | 1.14 | 0.024 | 1.07 | 1.00 | 1.13 | 0.035 |  |  |  |  | 0.31 |  |  |
|  |  | Waiting list | 4.45 (0.98) | 1.27 | 1.15 | 1.40 | 0.000 | 1.26 | 1.14 | 1.39 | 0.001 |  |  |  |  |  |  |  |
|  | Routine outcome measurement | Routine outcome measurement | 1.84 (1.53) | 1.09 | 1.02 | 1.17 | 0.006 |  |  |  |  |  |  |  |  | 0.30 |  |  |
|  |  | Use of routine outcome measurement | 1.66 (1.07) | 1.15 | 1.04 | 1.28 | 0.007 | 0.82 | 0.70 | 0.96 | 0.004 |  |  |  |  |  |  |  |
|  |  | HIC improvement-curve | 2.84 (1.48) | 1.05 | 0.98 | 1.13 | 0.124 |  |  |  |  |  |  |  |  |  |  |  |
|  | Reflection | Reflection | 3.29 (1.84) | 1.00 | 0.95 | 1.06 | 0.815 | 0.96 | O.91 | 1.01 | 0.169 |  |  |  |  | 0.30 |  |  |
|  |  | Education and training | 2.79 (1.78) | 0,.96 | 0.91 | 1.02 | 0.286 |  |  |  |  |  |  |  |  |  |  |  |
|  |  | Knowledge of FACT/ambulatory care | 3.63 (1.15) | 1.11 | 1.02 | 1.22 | 0.011 | 1.08 | 0.98 | 1.18 | 0.092 |  |  |  |  |  |  |  |
|  |  | Team spirit | 3.92 (1.17) | 1.14 | 1.05 | 1.24 | 0.001 | 1.13 | 0.04 | 1.23 | 0.005 |  |  |  |  |  |  |  |
|  | Mental health act | Carrying out mental health act | 4.05 (0.96) | 0..87 | 0.78 | 0.96 | 0.009 |  |  |  |  |  |  |  |  | 0.31 |  |  |
|  | Prot. | Protocol electronic health record | 3.37 (1.24) | 1.10 | 1.01 | 1.20 | 0.018 |  |  |  |  |  |  |  |  |  |  |  |
|  | building | Healing environment | 2.50 (1.25) | 0.92 | 0.85 | 1.00 | 0.053 |  |  |  |  |  |  |  |  | 0.39 |  |  |
|  |  | HC: individual rooms and bathrooms | 3.21 (2.02) | 0.90 | 0.86 | 0.95 | 0.000 | 0.91 | 0.86 | 0.97 | 0.002 | 1.09 | 1.02 | 1.92 | 0.044 |  |  |  |
|  |  | HC: comfort room | 3.84 (1.84) | 0.97 | 0.92 | 1.03 | 0.440 | 0.87 | 0.80 | 0.93 | 0.001 | 0.86 | 0.77 | 0.96 | 0.008 |  |  |  |
|  |  | HC: diversity of meeting spaces | 4.37 (1.48) | 0.91 | 0.84 | 0.98 | 0.023 |  |  |  |  |  |  |  |  |  |  |  |
|  |  | HC: family room | 2.47 (1.95) | 1.09 | 1.03 | 1.14 | 0.001 |  |  |  |  |  |  |  |  |  |  |  |
|  |  | HC: time-out/emergency bed | 2.89 (2.02) | 1.05 | 1.00 | 1.11 | 0.031 |  |  |  |  |  |  |  |  |  |  |  |
|  |  | HC: open workspace | 1.95 (1.72) | 1.07 | 0.99 | 1.15 | 0.058 |  |  |  |  |  |  |  |  |  |  |  |
|  |  | HC: domotics | 2.58 (1.98) | 0.88 | 0.83 | 0.92 | 0.000 | 0.93 | 0.87 | 0.99 | 0.028 |  |  |  |  |  |  |  |
|  |  | Intensive care (IC) | 2.26 (1.57) | 1.01 | 0.95 | 1.08 | 0.591 |  |  |  |  |  |  |  |  |  |  |  |
|  |  | Intensive care units (ICU) | 2.26 (1.57) | 1.08 | 1.01 | 1.16 | 0.024 |  |  |  |  |  |  |  |  |  |  |  |
|  |  | High Security Room (HSR) | 1.47 (1.18) | 0.77 | 0.70 | 0.84 | 0.000 | 0.57 | 0.49 | 0.68 | 0.001 |  |  |  |  |  |  |  |
|  |  | Healing environment |  |  |  |  |  | 0.99 | 0.97 | 1.02 | 0.529 | 0.96 | 0.92 | 0.99 | 0.025 |  |  |  |
|  | safety | Safety-management system | 3.97 (1.05) | 1.07 | 0.97 | 1.18 | 0.169 |  |  |  |  |  |  |  |  | 0.32 |  |  |
|  |  | Partnership agreement on safety | 3.61 (1.17) | 0.82 | 0.75 | 0.90 | 0.000 | 0.82 | 0.75 | 0.90 | 0.001 |  |  |  |  |  |  |  |
|  |  | Evaluation of coercion | 3.05 (1.41) | 1.06 | 0.98 | 1.14 | 0.101 |  |  |  |  |  |  |  |  | 0.32 |  |  |
|  |  | Argus feedback | 3.18 (1.52) | 0.96 | 0.89 | 1.04 | 0.366 |  |  |  |  |  |  |  |  |  |  |  |
|  |  | Total score | 189.5 (29.1) |  |  |  |  |  |  |  |  |  |  |  |  | 0.27 |  |  |
